# The Impact of Education on Fertility During the Chinese Reform Era (1980–2018): Changes Across Birth Cohorts and Interaction with Fertility Policies

**DOI:** 10.1007/s10680-023-09691-2

**Published:** 2024-01-30

**Authors:** Pau Baizan, Wanli Nie

**Affiliations:** 1 Institució Catalana de Recerca i Estudis Avançats (ICREA), 23 Passeig de Lluís Companys, 08010 Barcelona, Spain; 2 Department of Statistical Sciences Paolo Fortunati, University of Bologna, Via Belle Arti, 41, 40126 Bologna, Italy; 3 Department of Political and Social Sciences, Universitat Pompeu Fabra, Barcelona, 25 Ramon Trias Fargas street, 08005 Spain

**Keywords:** Fertility, Education, China, Social institutions, Life course, Event history analysis

## Abstract

We examined the influence of education on fertility decisions in contemporary China, drawing upon theoretical insights that emphasise the role of social institutions, gender relations, and life course dynamics in shaping family behaviour. This led us to propose a set of hypotheses that explain the differential effect of education on each parity. We used information on female cohorts born between 1960 and 1989, coming from the China Family Panel Studies for 2010–2018. We applied event history models with both independent and simultaneous equations models to account for selection and endogeneity effects. The results point to a substantial contribution of the increased educational attainment in the population in the fertility decline and current low levels of fertility, beyond the role of fertility policies. Consistent with our hypotheses, the results show that woman’s educational attainment has a strong negative effect on the hazard of bearing a second or third child*.* Male partner’s educational attainment also has a negative effect on the hazard of transition to a second or third birth, yet with a weaker intensity. We also found that the negative effect of education on second birth rates significantly declines across birth cohorts. The results show little educational differentials in the probability of bearing a first child, while the better educated postpone first births. Moreover, the effect of fertility policies, measured at the individual level, gradually increases with the level of education.

## Introduction

China’s road to below replacement fertility was accomplished in an astoundingly short period of time. Most of the decline was completed during the 1970s, from a total fertility rate of 5.8 in 1970 to 2.7 in 1979. During the 1980s, fertility levels fluctuated slightly above 2 children per woman and dropped to below replacement from the early 1990s. There is a consensus among different sources in estimating a TFR of around 1.5 children per woman since the mid-1990s (Feng, 2015). Several scholars have highlighted the role of massive socio-economic changes in explaining this decline and the diminishing role of family planning policies (Cai, 2010; Feng, 2015; Zhao & Zhang, 2018). Indeed, the end of the one-child policy in 2016 did not substantially alter fertility levels (Gietel-Basten, 2019).

Parallel to these fertility trends, there have been substantial changes in the Chinese institutional context. The institutional configuration of society, including family policies, has been linked to fertility levels by an expanding theoretical literature (Esping-Andersen, 1999; Huinink et al., 2015; McDonald, 2000; McNicoll, 1994; Rindfuss and Choe 2016; Thévenon & Neyer, 2014). These authors highlight how the state, market, and families interact to provide welfare for individuals and families, with broadly predictable consequences for fertility levels. Key dimensions of the institutional environment are the gender system and the social mobility system (Goldscheider et al., 2015; Greenhalgh, 1988). Our framework also emphasises the presence of cultural influences in family behaviour, including both long-term continuities such as the importance of kinship and intergenerational relationships, as well as innovations such as the strength of dual-breadwinner couples and the rise of the “quality child” (Greenhalgh & Winckler, 2005; Pfau-Effinger, 2005). One way to assess institutional influences is through their differential effects by socio-economic position of individuals, and more specifically their educational level, which can be seen as a proxy for socio-economic status. Individuals and families with different educational levels are subject to differing constraints and incentives for fertility and are likely to hold different cultural views. Evaluating the relationship between education and fertility is, therefore, crucial to understanding the individual-level mechanisms that explain recent very low fertility levels. Previous literature has shown that this relationship is highly context-specific, and that it changes over the demographic transition (Coale & Watkins, 1986; Klesment et al., 2014; Lutz & Skirbekk, 2014). The literature on education and fertility in China suggests that the educational differentials are substantial and that these differentials have widened in the early stages of the demographic transition, i.e. the cohorts born between the 1930s and the 1960s (Lavely & Freedman, 1990; Niu & Qi, 2020; Piotrowski & Tong, 2016). Yet, the individual level relationship between education and fertility has not been carefully investigated for more recent birth cohorts. Most existing analyses use aggregate-level measures and cross-sectional data or focus on particular regions (Feeney and Wang Feng 1993; Lan & Kuang, 2016; Zhang, 1990). They mainly investigate the effects of contextual variables related to socio-economic development, such as GDP growth, urbanisation and birth-control policies (Niu & Qi, 2020). Only a few studies use longitudinal individual-level data, but they refer to the period up to the 1980s, when the central stages of the fertility transition took place (Piotrowski & Tong, 2016). Previous studies did not control for the effects of fertility policies at the individual level. This control is important because, given the design of the policies, they are likely to have differential effects by educational group, as argued below. Moreover, previous studies did not control for key cofounders of the relationship education-fertility, such as family background variables.

Here the aim is to evaluate the effect of education on fertility behaviour at the individual level for the cohorts born from 1960 to 1989, who were in the childbearing stage during the period of political and economic reforms that started in the late 1970s (the Reform Era), which includes the later stages of the demographic transition. We adopt a life course approach and conduct specific analyses by birth order, accounting for the effect of a wide array of key variables, including fertility policies measured at the individual level (Elder et al., 2003; Huinink & Kohli, 2014). We focus the analyses on woman’s educational trajectories and fertility, although we also include analyses of the male partner’s level of education. Moreover, we pay attention to changes over birth cohorts in the effect of education on fertility, as well as possible interaction effects with fertility policies. Through the use of event history analyses, we can evaluate time-dependent dynamics for first, second and third order births, including the cohorts of women who have not yet completed their reproductive lives. Previous literature has shown the need to account for selection effects to properly assess the effect of education on fertility (Kravdal, 2001). In addition, fertility and educational attainment may be affected by unobserved factors common to both processes, such as social mobility aspirations or familistic values. As a modelling strategy, we adopt a simultaneous equations approach to test the presence of endogeneity between education and fertility (Lillard, 1993; Upchurch et al., 2002).^1^

The remainder of the paper is organised as follows. In section two we give a brief overview of the theoretical arguments on the education-fertility relationship. In section three we review several features of the Chinese institutional configuration and policies, linking them to the specific constraints and incentives for fertility for each educational level. This leads us to propose a set of hypotheses that explain the differential effect of education on each parity. Section four deals with the data and methods used in our analyses. In section five, we present both descriptive results and multivariate results, including a comparison of models using standard event history techniques with models using simultaneous equations. The final section provides some concluding remarks and reflections.

## Theoretical perspectives on the relationship education-fertility

Education plays a key role in many theories explaining fertility levels and their changes over time (Bongaarts, 2010; Kravdal & Rindfuss, 2008; Lutz & Skirbekk, 2014). While a comprehensive account of the theories linking education and fertility is beyond the scope of this paper, we will highlight the most prominent mechanisms proposed by the theoretical approaches that underpin our hypotheses and analyses, i.e. microeconomics, Caldwell’s wealth flows theory, gender equity, and institutional perspectives.

Microeconomic theory links fertility decisions to household economic processes, such as labour force participation and consumption (Becker, 1991). A basic proposition is that the parents’ demand for children is, in fact, a demand for the services that children provide over time, which may include labour, old age security, and “consumption” utility (Robinson, 1997). On the costs side, it is emphasised that the price of children includes foregone women’s wages and career opportunities linked to the care of children. These opportunity costs are higher for the better educated, due to their higher earning potential. The demand for children is positively affected by household income. As a result, a high income should stimulate fertility, leading to the expectation that better-educated men should have a higher number of children. Yet, this income effect may be offset by the increase in the parental resources spent on each child linked to a higher income, i.e. to the child’s “quality”. This is particularly likely in contexts where most of the cost of children (especially educational costs) fall on parents. Overall, the microeconomic theory provides a framework for investigating fertility at the household level, but as such is silent about the contextual and institutional conditions that change costs, income, and preferences, and thereby fertility decline. Additional contributions, however, point out that the key factors leading to a change from a high fertility equilibrium to a low fertility equilibrium are an increase in the returns to education, together with an increase in real wages (Becker et al., 1990).

Caldwell’s intergenerational “wealth flows” theory also focuses on children’s costs (Caldwell, 1980, 2005). He highlights the importance of children as economic assets over the parents’ life course in settings where family production (especially subsistence agriculture) prevails, creating incentives for a large family. The positive flow of resources from children to parents is reversed by the introduction of mass schooling in a society, which sharply increases the costs of children. Caldwell’s theory also emphasises that education conveys new values that undermine parental influence over children, favouring children’s independence, and destabilising the traditional family economic structure. All these influences reduce the value of children to parents, leading to lower fertility.

Gender approaches to fertility emphasise the role of the changes in institutions and social structure, particularly concerning the labour market and family organization (Goldscheider et al., 2015; Mason, 1997a). McDonald (1997, 2000) argues that the fertility transition is associated with an increase in gender equity inside the family, linked to a change in the “family morality”, fueled by increased educational levels, declining infant mortality, and the availability of family planning services. Moreover, when a majority of women participate in individual-oriented institutions, such as education and the labor market, very low fertility levels are reached if women continue to take the primary responsibility for the care of children. Only when family-oriented institutions, including family policies, industrial relations, and the family itself become more gender-equitable, can fertility approach replacement levels. At the individual or family level, the relevant mechanisms are the opportunity costs borne by women and gender-role ideologies, which are shaped by the configuration of institutions and the dominant cultural norms existing in a society.

More generally, institutional approaches to fertility focus on the political, economic, and institutional context within which demographic decision-making takes place (McNicoll, 2001; Rindfuss and Choe 2016). Each institutional configuration has different consequences for gender and socio-economic stratification, impinging on the influence of education on fertility. Several institutions are key for fertility, including the family and the local public administration, the stratification system and the mobility paths that it accommodates, the labor market, the school system, and welfare and fertility policies (Greenhalgh, 1988; Hoem, 2008; McNicoll, 1994). Different combinations of these factors and institutions lead to highly idiosyncratic and historically contingent demographic transitions, as well as post-transitional fertility trends (Mason, 1997b). Despite this diversity, it is generally found that in the first half of the demographic transition socio-economic differentials in fertility widen, leading to a negative education-fertility relationship (Coale & Watkins, 1986; Piotrowski & Tong, 2016).

Finally, education has been found to lead to a postponement in the timing of childbearing (Blossfeld & Huinink, 1991; Gustafsson, 2001). The postponement of first births results, on the one hand, from the difficulty in combining the roles of parent and student and, on the other hand, from the subsequent delay in the adoption of adult roles, such as integration in the labour market and marriage.

## The Chinese institutional context (1980–2018) and the educational stratification of fertility

The economic and social policy reforms that started in the late 1970s are at the origin of the contemporary welfare model. The transition to the market economy involved a gradual reduction of the state sector and provision of welfare, involving a complete shift in the costs of children from the collective to the family (Brandt & Rawski, 2008; He & Wu, 2021). Agriculture was de-collectivised during the first years of the Reform, making the family the core unit of production and welfare (Greenhalgh & Winckler, 2005). At the same time, this period of accelerated economic growth and urbanisation brought about new opportunities for upward economic and social mobility for individuals and families. Ever-increasing investments in education became necessary to successfully compete in the labour market and take advantage of the rise in the returns to education (Zhang & Zhao, 2007). The increase in educational attainment can be illustrated with data from the China Family Panel Study for the birth-cohorts studied here. During the 1960s and 1970s, the focus was placed on basic education, which still did not reach the whole population. As a consequence, the oldest birth-cohorts studied here could only partially benefit from the expansion of the educational system to the secondary and tertiary levels of education, since most of their childhood occurred before the onset of the Reform Era. 29 percent of women born during 1960–69 did not reach a primary level of education, while this was the case for less than 5 percent for the 1980–89 birth-cohort. For the same female birth-cohorts, tertiary education increased from less than 1 percent to about 15 percent (Table 1). These data also show that the gender gap in education has almost disappeared.

**Table 1:** Educational attainment of the birth-cohorts 1960–69, 1970–79, and 1980–89 (in percentage)

Birth-cohort	1960–69		1970–79		1980–89	
	Women	Men	Women	Men	Women	Men
Educational level						
Less than primary	29.1	13.1	20.3	10.6	4.9	5.0
Primary	24.2	24.7	24.8	21.2	11.3	10.7
Lower secondary	28.8	37.5	31.4	39.0	37.7	36.0
Higher secondary	17.0	22.2	18.8	24.2	31.1	34.1
Tertiary	0.9	2.5	4.6	5.0	15.0	14.2
Total	100	100	100	100	100	100
Sample size (weighted)	2,617	2,454	2,494	2,152	2,000	1,747

Based on data from the Chinese Family Panel Studies 2018. Weighted data

Values emphasising the “quality child” and an intensive involvement of mothers in their child(ren)’s education and care are widely prevalent in contemporary China (Greenhalgh, 2008). In a context with intense educational competition, heightened investments in education are needed to secure social mobility, irrespective of the parental social position. But parenting strategies and aspirations that emphasise providing high-quality resources are likely to be more prevalent among more educated parents, not least because of the higher availability of resources linked to social class and because more investments are needed to increase or maintain (relative) parental social level across generations (Breen & Goldthorpe, 1997). More educated parents, therefore, should be more likely to concentrate their resources on one child and only exceptionally bear a second or third birth, consistently with Becker’s hypothesis of an interaction between quality and quantity (Becker, 1991; Becker & Lewis, 1973). The existing high level of educational and social homogamy between partners should reinforce this effect (Hu, 2016; Hu & Qian, 2016).

The link between the fate of children and their parents is reinforced by the persistence of a “strong family” culture that emphasises the importance of vertical kinship relationships and family continuity (Chen & Li, 2014). The close social and economic interdependence between generations over the life course includes the provision of care and material support from children to parents in old age, linking children’s to parent’s economic position. As a result, parental investments in their children’s education directly benefit parents in the long run. The analyses of intergenerational transfers show that the Chinese elderly rely on family resources to a substantial extent (Lee, 2013). While a high degree of interdependence between generations prevails among all social groups, it is likely to be stronger for the low educated. This is particularly so among agricultural families, for which child’s labour and support became crucial for the family’s economy, especially during the early Reform years (Caldwell, 2005; Greenhalgh & Winckler, 2005). Moreover, lower socio-economic status families have lower access to public pensions and have a lower saving capacity, providing incentives for additional births. The reliance on children by the low educated is enhanced by the lack of economic security and the need to diversify sources of income (e.g. by migrating to urban areas). As a result, the low educated should show higher fertility levels.

The strong interdependence between generations involves that parental obligations are also substantial. Care from grandmothers is essential to allow a minimum of compatibility between women’s jobs and childrearing in a context where the majority of women with low-age children are full-time employed (Dasgupta et al., 2015).^2^ It is remarkable that the statutory retirement age for women is 50 years (for female public servants, it is 55; and 60 for men), allowing them to participate in childcare. The Chinese welfare regime, therefore, blends ample opportunities for career advancement, also for women, with several typical characteristics of the “unsupported” familistic model (Ferrera, 2013; Leitner, 2003; McDonald, 1997). Full-time labour market participation of women is expected, but compatibility with mother roles has been increasingly difficult, given the lack of formal childcare availability and despite grandmothers’ help (Jinglun & Xin, 2020; Zhong & Peng, 2020). The pre-reform comprehensive family support system, based on the work units (“danwei”), provided childcare and other social services, allowing a high level of compatibility between women’s employment and family obligations. The gradual retreat of collective and state-owned enterprises, together with the increasing marketization, meant that family responsibilities were shifted back to parents (He & Wu, 2021). The gap in care is especially acute between the end of maternity leave^3^ (of about 3–6 months) and the start of education at age 3 or 6 of the child. Leave arrangements reflect deeply gendered cultural conceptions about gender roles (Brinton and Lee 2016). Women’s career advancement is highly compromised by bearing a child, especially a second child, as women fear discrimination by employers (Zhou, 2019). Several studies have shown an increased level of gender segregation of occupations and earnings inequality (Bauer et al., 1992; He & Wu, 2018). Statistical gender discrimination is further reinforced by the early age of retirement for women which discourages skill investments from employers to their female employees, as the investments will be used for a shorter period of time. Highly educated women are especially likely to be hit by discriminatory practices, because they have higher returns to experience and job tenure than lower-educated women, and therefore any interruption in employment associated with motherhood results in stronger income penalties (England et al., 2016). Conversely, for lower-educated women, labour market interruptions involve a lower penalty in terms of future earnings and the probability of returning to an equivalent job if they leave the labour market to take care of a child. The resulting differential in opportunity costs of childbearing by women’s level of education provides an additional argument to expect strong educational stratification in second and third births.

The above discussion has highlighted the expected differentials in childbearing costs and child’s “quality” by women’s level of education, based on microeconomic explanations of fertility. We have also argued that low-educated couples had stronger incentives to have larger families than better-educated couples, consistently with Caldwell’s contention that children are an economic asset for the former (Caldwell, 1980). Finally, we have put forward several features of the social-institutional context that, according to McDonald’s “gender equity” theory, discourage childbearing particularly among the better-educated (McDonald, 2013). Overall, these arguments lead us to propose that *women’s educational attainment has a strong negative effect on the hazard of transition to a second or third child (Hypothesis 1)*.

Note that the arguments presented concerning the fertility effects of parental investments in child quality and the role of intergenerational relations, together with a strong educational homogamy in couples, apply to both men’s and women’s fertility. It can be hypothesised that, in the context studied, these factors are likely to prevail over the positive effect of men’s income. Therefore, we propose that *male partner’s educational attainment has a negative effect on the hazard of transition to a second or third birth (Hypothesis 2).*

Some of the factors favouring a strong interdependence between generations, noted above, may have weakened over time, because of the decline in household production and the growing economic independence of children and women, potentially leading to a reduction of the educational fertility differentials. Market reforms were selectively applied to agriculture since 1979, and only eventually were gradually applied to the rest of the economy (Brandt & Rawski, 2008). Therefore, the incentives for bearing several children were highest among agricultural families (mostly low educated) during the initial Reform years, to decline subsequently, as a result of the improvements in productivity and the emergence of alternative economic opportunities. Moreover, the fast increase in educational opportunities, especially at the secondary and tertiary levels, is consistent with a generalized switch to the “quality” child. But perhaps the most powerful force potentially leading to convergence across educational levels is the increase in the economic returns to education and the associated social mobility, linked to the expansion of the market economy. This trend enhanced the incentives to invest in education for the whole population, further reducing childbearing incentives for the low educated. At the same time, higher returns to education boosted the incomes of the better educated, thus lessening their childbearing costs. Yet, the gradual marketization of family support services, especially since the mid1990s, may have increased the opportunity cost of childbearing for the better-educated, possibly countering part of the reduction in educational differentials over time. An additional argument in support of the inter-generational convergence between educational groups in fertility behaviour is the spread of fertility norms favouring the one-child family (Zheng et al., 2009, 2018). The factors just mentioned, in particular the expansion of education, largely follow a generational pattern, leading us to propose that, overall, *the negative effect of education for second and third births rates declines across birth cohorts (Hypothesis 3).*

Economic needs as well as normative pressure work in tandem for family continuity, providing incentives for marriage and bearing at least one child (Zhou, 2019; to 2013). Marriage is still practically universal, although there are some limited signs of increasing diversification of the partnership formation process, including unmarried cohabitation and premarital conceptions (Ma & Rizzi, 2017). Childbearing outside marriage remains rare (Raymo et al., 2015a, 2015b). Marriage offers opportunities for income and status enhancement, especially for women, in a context with sizeable gender gaps in education and income and where two incomes are necessary for households’ economic sufficiency (Shu & Bian, 2003). Yet, marriage also involves a strong normative pressure to have one child shortly after marriage, together with other family obligations (Jones, 2007). Values emphasising the importance of motherhood are widely prevalent (Gu, 2009). Moreover, family polices never questioned first births but instead promoted and even idealised the two-parent family with one child (Greenhalgh & Winckler, 2005). In this context, it can be expected that most women bear at least one child. At the same time, life course studies have shown the delaying effect of education on the timing of childbirth (Blossfeld & Huinink, 1991; Ní Bhrolcháin & Beaujouan, 2012; Oppenheimer, 1988). From the above arguments we derive hypothesis 4: *Irrespective of the educational level, most women bear at least one child. The effect of education on first childbearing is mainly limited to its postponement by the highly educated*.

Fertility policies are, of course, an essential component of the regime package, which has been thoroughly studied (e.g. Gu et al., 2007). The policy prescriptions have greatly varied over time, which allowed for a different number of children and a range of conditions under which one, two, or exceptionally three births were allowed for particular couples (single mothers are subject to paying a “social supporting fee”). The “Later-longer-fewer” period from 1971 to 1980 greatly boosted contraception and late marriage. Its prescriptions included.

1. Later marriage, which means a minimum marriage age of 25 for males and 23 for females,
2. Longer birth intervals, of at least 4 years between two births, and
3. fewer children, or at most two children.

The strict “one-child policy”, introduced in 1980, was initially resisted in rural areas, where state control was weakest and the economic and social benefits of several children were more evident (Greenhalgh & Winckler, 2005). Lack of compliance and difficulties in imposing the new regulations prompted an adaptation of the policies to the socio-economic circumstances of families, especially since the mid-1980s. Thus, 2 or even 3 children were allowed in the case of agricultural families (for instance, if the first or first two children were girls), while the one-child norm was strictly imposed in economically advanced areas (Zeng, 1989). The “political costs” of having a child not allowed by the policy were probably higher for better educated couples. Such economic and social penalties may include obstacles in career advancement, access to housing, or the lack of “hukou” registration for the beyond-quota child and its associated benefits, such as access to public schooling. Sanctions could be more readily applied in urban areas and particularly to state sector employees. As a result, conforming to the policy conferred economic and social benefits that were positively stratified by the level of education. The gradual loosening of the policy led to the adoption of a comprehensive “two-child policy” in 2016. The discussion just presented highlights that the effect of fertility policies must be accounted for to properly estimate the impact of education on fertility. Moreover, it implies that the effect of education on fertility was moderated by the family planning policies, leading us to propose that *the negative effect of family planning policies on fertility was stronger for the highly educated* (Hypothesis 5).

## Data and methods

### Data

The data sets that we used are from the China Family Panel Studies^4^ (CFPS) for 2010–2018 (Institute of Social Science Survey, Peking University, 2015). The first wave of the CFPS was designed as a nationally representative sample of the population of the People’s Republic of China living in private households in 2010 (Xie & Lu, 2015). Almost 15,000 families and 30,000 individuals within these families were interviewed, with an approximate response rate of 79 percent. These original sample members were reinterviewed every 2 years and, if they split off from their original households to form new households, all adult members of these new households were also interviewed. Similarly, children in the original sample households were interviewed when they reached 9 years of age. In addition to providing information on respondents within the panel survey period (2010 onwards), the CFPS asked respondents to provide detailed retrospective fertility histories. These retrospective data were matched to the within-panel data to construct detailed fertility histories from age 15 years for all adult female respondents.

We used information on female birth-cohorts from 1960 to 1989, which consisted of an initial sample of 15,086 women. To avoid possible bias due to correlation between the responses of women belonging to the same household, we randomly selected a woman in each household with more than one eligible female respondent, leading to the exclusion from the sample of 2264 women. We also excluded from the study sample respondents who gave birth below age 15. We kept in the analyses one twin (or triplet) birth only. The final analytical sample included 12,822 first birth episodes, 11,766 s birth episodes, and 6396 third birth episodes, belonging to 12,838 women (Table 2).

**Table 2:** Analytical sample’s descriptive statistics

	Proportion	s.e
Woman’s education		
Less than primary	0.224	0.004
Primary	0.204	0.004
Low secondary	0.296	0.004
High secondary	0.141	0.003
Tertiary	0.134	0.003
Missing*	0.000	
Mothers’ education		
Less than primary	0.579	0.004
Primary	0.227	0.003
Low secondary	0.131	0.003
High secondary or tertiary	0.063	0.002
Missing*	0.184	
Parental occupation		
Agriculture	0.547	0.004
Unskilled	0.064	0.002
Skilled	0.085	0.002
Services	0.153	0.003
Professional	0.151	0.003
Missing*	0.411	
Birth-cohort		
1960–69	0.358	0.003
1970–79	0.337	0.003
1980–89	0.305	0.003
Missing*	0.000	
Siblings: No siblings	0.096	0.003
1 sibling	0.192	0.003
2 + siblings	0.712	0.003
Missing*	0.321	
Hukou registration: Rural	0.843	0.003
Urban	0.157	0.003
Missing*	0.162	
Parent communist party member: yes	0.183	0.003
No	0.812	0.003
Missing*	0.357	
Ethnic minority: yes	0.092	0.003
No	0.908	0.003
Missing*	0.087	
No. of first birth episodes	12,822	
No. of second birth episodes	11,766	
No. of third birth episodes	6,396	
No. of first births	11,777	
No. of second births	6,310	
No. of third births	1,412	
No. women	12,838	

*Source* China Family Panel Studies 2010–2018. Unweighted data. *percentage with missing information. The percentages of the variables include imputed cases

The events of first, second, or third conception leading to a birth are indicated by the date of the birth, given to the nearest month, minus 9 months. For first births, observation begins at age 15 and ends with the event of the conception of the first child or, for right-censored cases, with the date of the interview or by reaching age 45, whichever comes first. Similarly, the episodes of second and third births start the month just after previous birth and end with the event of conception or with censoring (interview date or 15 years after previous birth).

**Fig. 1:**
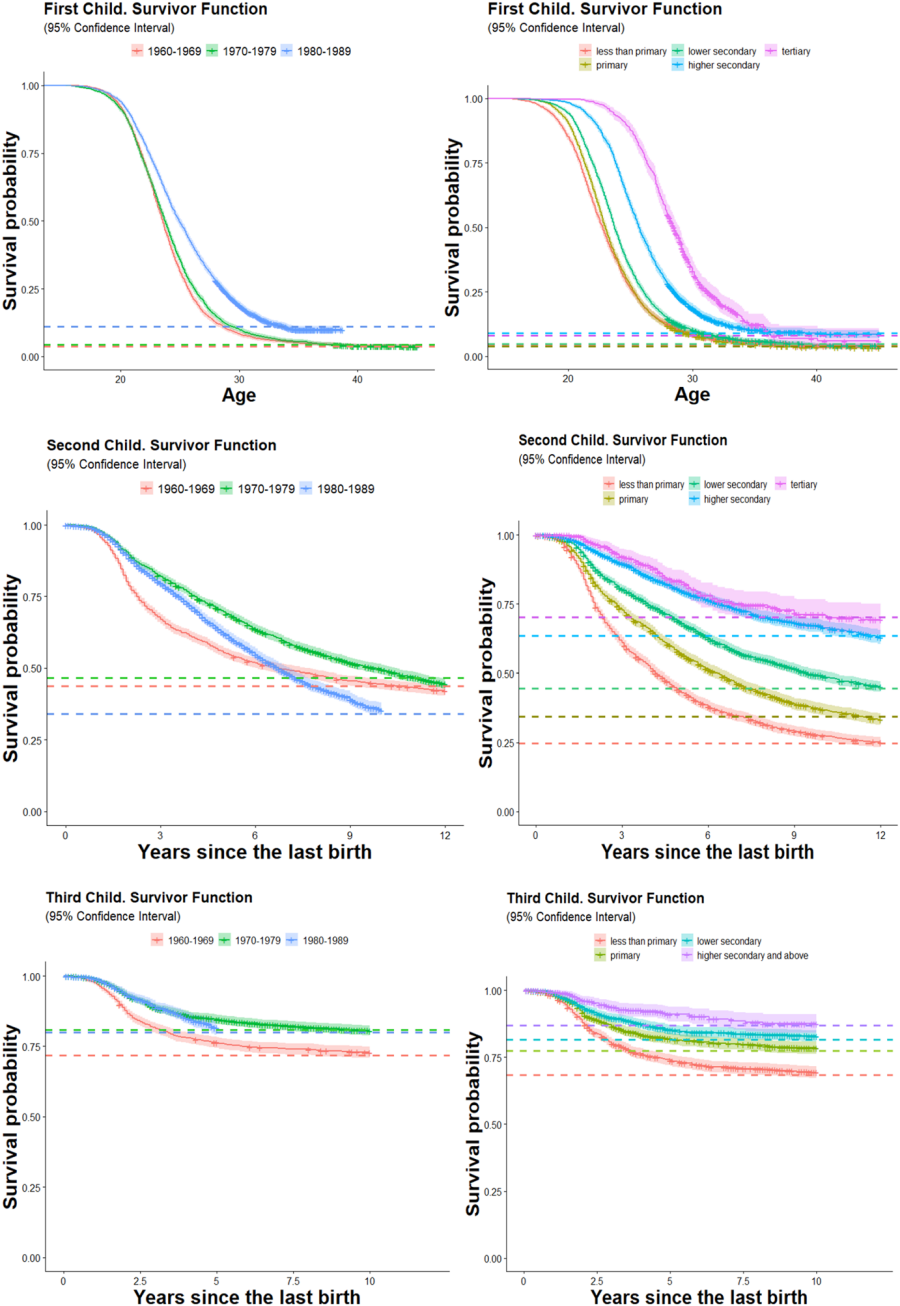
Survivor function of the first, second and third births by women’s birth cohort (left graphs) and highest obtained education level of the woman. The survivor function is based on the birth date of the children rather than the conception date

The survey does not provide detailed educational histories but contains information on the educational level attained at each survey wave. Thus, to construct educational histories, we assumed that women were enrolled in each level of education up to the minimum age required to attain that level and updated the level of education accordingly. This assumption is unlikely to affect the results (especially in terms of reverse causation) since only a few individuals have children before their age at finishing education (Fig. 1). To test hypothesis 2 we performed analyses including the male partner’s level of education for second and third birth episodes. This variable refers to the men’s educational level at the beginning of the episode for married and unmarried couples. These analyses exclude periods in which the women were not in a partnership.

Previous studies have shown that family background factors independently influence both education and fertility (Axinn et al., 1994; Chen, 2020; Kan & Hertog, 2017; Yi et al., 2015). Moreover, the values and goals learned during childhood, the social environment, and the economic resources available in the parental home can act as common factors influencing fertility and educational behaviour (Nisén et al., 2014; Tropf & Mandemakers, 2017). The CFPS is rich in indicators about the respondents’ family of origin, including her mother’s educational level, the respondent’s number of siblings, the type of residence during childhood (rural or urban Hukou registration), the parental political status (whether at least one of the parents was a member of the communist party during the respondent’s childhood), and the occupational status of the family of origin. The occupational status of the family reports the highest occupation between the parents when the respondent was 14 (Table 2). All the above-mentioned information is estimated at the latest wave, to correspond to the most complete life history available. To control for family policy effects and test hypothesis 5 we constructed a fertility policy variable indicating whether a birth was allowed. This time-varying variable accounts for the policies formally applying to each woman, considering her marriage and fertility history, province of residence, ethnicity, rural/urban residence, gender of previous child(ren), her and her partner’s number of siblings, and time period. We assigned values to the explanatory variables with missing information using a multiple imputation technique (Honaker et al., 2011).

### Statistical methods

We apply event history methods to analyse the impact of education on fertility for first, second and third birth conceptions (Tables 3 and 4). The main effects of these models allow us to assess the effects of women’s education (Hypothesis 1), men’s education (Hypothesis 2), as well as differential timing of births by educational level (Hypothesis 4). To properly assess the effects of education, we control for several variables including age, duration since previous birth, birth cohort, family background and fertility policy. Additionally, we estimated a model including an interaction between education and birth cohort to test Hypothesis 3 on the possible differential effect of education by birth cohort (Table 5). Similarly, to test the possible differential effect of policies by level of education we estimated a specific model that interacts these variables (Table 6).

Yet to identify the impact of education on fertility we need to disentangle it from the potential existence of selection effects. More specifically, we must account, on the one hand, for the unobserved factors affecting fertility, and on the other hand, for the possible unobserved factors influencing fertility and education simultaneously. Below we explain in detail our analytical strategy to account for selection effects and the rationale for the methods used. A first step involves the use of separate hazard models for the processes of first, second, and third birth conception (Model 1). This can be represented mathematically in the following way (Lillard and Panis 2003):

$${\mathit{ln }h}_{i}^{1B}\left(t\right)={\beta }_{0}^{1B}+{\beta }_{1}^{1B\prime}{T}_{i}\left(t\right)+{\beta }_{2}^{1B\prime}{Z}_{i}\left(t\right) +{\beta }_{3}^{1B\prime}{X}_{i}(t)$$

$${\mathit{ln }h}_{i}^{2B}\left(t\right)={\beta }_{0}^{2B}+{\beta }_{1}^{2B\prime}{T}_{i}\left(t\right)+{\beta }_{2}^{2B\prime}{Z}_{i}\left(t\right) +{\beta }_{3}^{2B\prime}{X}_{i}(t)$$

$${\mathit{ln }h}_{i}^{3B}\left(t\right)={\beta }_{0}^{3B}+{\beta }_{1}^{3B\prime}{T}_{i}\left(t\right)+{\beta }_{2}^{3B\prime}{Z}_{i}\left(t\right) +{\beta }_{3}^{3B\prime}{X}_{i}(t)$$

where ln *h*_*i*_(*t*) is the log-hazard of occurrence of a birth at time *t* for woman *i* and *1B*, *2B* and *3B* are symbols for first, second and third births, respectively. In these equations ${\beta }_{0}$ is a constant, *T* denotes a piecewise linear spline that captures the baseline effect of duration on intensity, $Z$ is a vector of dummies for educational categories and *X* represents a vector of other (potentially time-varying) covariates. Model 2 additionally includes interactions between education and age (for first births) and between education and duration since previous birth (for second and third births). These interactions account for the different timing of births by education. Their inclusion in the models allows testing whether the better educated postpone first births (Hypothesis 4) and facilitates the comparison of the effects between educational groups as they are net from timing effects.

The specification above, however, does not consider the possible existence of selection effects linked to the unobserved heterogeneity in the population in the propensity to bear a child. For instance, some woman’s unobserved characteristics, such as a greater propensity towards building a career as opposed to a family or primary infecundity, may systematically lead to lower fertility. Familistic attitudes and the greater economic advantages of fertility for the household economy (e.g. agricultural households) are likely to lead to higher fertility. Previous research has shown that these biases can be corrected by using simultaneous equations for first, second, and third births, in which a common heterogeneity term is added to each birth equation (Kravdal, 2001). The three fertility equations are modeled jointly, using a common unobserved residual ${\varepsilon }_{i}$ reflecting unobserved woman-specific constant factors influencing all her births (Models 3 and 4). The statistical specification of the hazard models is otherwise identical as in Models 1 and 2 above.

$${\mathit{ln }h}_{i}^{1B}\left(t\right)={\beta }_{0}^{1B}+{\beta }_{1}^{1B\prime}{T}_{i}\left(t\right)+{\beta }_{2}^{1B\prime}{Z}_{i}\left(t\right) +{\beta }_{3}^{1B\prime}{X}_{i}\left(t\right) +{\varepsilon }_{i}$$

$${\mathit{ln }h}_{i}^{2B}\left(t\right)={\beta }_{0}^{2B}+{\beta }_{1}^{2B\prime}{T}_{i}\left(t\right)+{\beta }_{2}^{2B\prime}{Z}_{i}\left(t\right) +{\beta }_{3}^{2B\prime}{X}_{i}\left(t\right) +{\varepsilon }_{i}$$

$${\mathit{ln }h}_{i}^{3B}\left(t\right)={\beta }_{0}^{3B}+{\beta }_{1}^{3B\prime}{T}_{i}\left(t\right)+{\beta }_{2}^{3B\prime}{Z}_{i}\left(t\right) +{\beta }_{3}^{3B\prime}{X}_{i}\left(t\right) +{\varepsilon }_{i}$$

A second type of potential bias may arise if unmeasured attributes affect both educational attainment and fertility. Educational attainment goals and strategies might not be exogenous to fertility choices, as these two roles compete in time and resources (Huinink & Kohli, 2014). Unmeasured attributes such as health status, social mobility aspirations, or familistic values may affect both fertility and educational attainment, potentially biasing the estimated effect of education on fertility. To investigate whether there is a joint determining effect for both processes, we run a multi-process model of educational attainment and fertility. The statistical specification is derived from the framework developed by Lillard (1993), Upchurch et al. (2002), andKravdal (2001).^5^ It consists of four simultaneous equations, three of them specified as event history models for first, second and third birth conceptions, and an additional probit equation for the individual’s progression to the next educational level (Lillard and Panis 2003) (Models 5 and 6 in annex).

$${\mathit{ln }h}_{i}^{1B}\left(t\right)={\beta }_{0}^{1B}+{\beta }_{1}^{1B\prime}{T}_{i}\left(t\right)+{\beta }_{2}^{1B\prime}{Z}_{i}\left(t\right) +{\beta }_{3}^{1B\prime}{X}_{i}\left(t\right) +{\varepsilon }_{i}$$

$${\mathit{ln }h}_{i}^{2B}\left(t\right)={\beta }_{0}^{2B}+{\beta }_{1}^{2B\prime}{T}_{i}\left(t\right)+{\beta }_{2}^{2B\prime}{Z}_{i}\left(t\right) +{\beta }_{3}^{2B\prime}{X}_{i}\left(t\right) +{\varepsilon }_{i}$$

$${\mathit{ln }h}_{i}^{3B}\left(t\right)={\beta }_{0}^{3B}+{\beta }_{1}^{3B\prime}{T}_{i}\left(t\right)+{\beta }_{2}^{3B\prime}{Z}_{i}\left(t\right) +{\beta }_{3}^{3B\prime}{X}_{i}\left(t\right) +{\varepsilon }_{i}$$

$${E}_{ij}^{*}={\beta }_{0}^{E}+ {\beta }_{1}^{E\prime}{X}_{ij}+{\lambda }_{i}+{u}_{ij}$$

The three fertility equations are specified as in Models 3 and 4 above, in which the random variable ε reflects unobserved woman-specific constant factors influencing births. Educational attainment is specified as a multilevel probit model where each woman makes one or more educational decisions (attaining, or not, each subsequent level of education). Educational decisions are nested within women. Each woman may make up to 5 educational decisions, corresponding to the attainment of the following educational levels: primary, lower secondary, higher secondary, college, and university degree. Each educational decision is conditional on the attainment of the previous level of education. This operationalization is consistent with the conceptualization of education as a life course trajectory. ${E}_{ij}^{*}$ indicates the latent propensity that a woman *i* attains level *j* (*j* = 1,…5). If ${E}_{ij}^{*}$ < 0, the woman does not attain a particular level of education (*E*_*ij*_ = 0), and if ${E}_{ij}^{*}$ ≥ 0, the woman attains that level (*E*_*ij*_ = 1). Observed characteristics are captured by the set of regressors X_ij_.^6^ Unmeasured characteristics are in part woman-specific and constant across all her educational decisions (*λ*_*i*_) and in part specific to each educational decision for each level of education (*u*_*ij*_). In Models 5 and 6, the random variables *ε* and *λ* are assumed to follow a joint bivariate normal distribution:

$$\left(\begin{array}{c}\varepsilon \\ \lambda \end{array}\right)\sim N\left(\begin{array}{cc}\left(\begin{array}{c}0\\ 0\end{array}\right),& \left(\begin{array}{cc}{\sigma }_{\varepsilon }^{2}& \\ {\rho }_{\varepsilon \lambda }& {\sigma }_{\lambda }^{2}\end{array}\right)\end{array}\right)$$

where ρ_ελ_ represents the correlation between the unobserved heterogeneity terms of the processes of fertility and educational attainment. This correlation provides a test of whether women with unobserved above-average risks of fertility (ε > 0) also tend to have below-average educational attainment propensities (λ < 0) and vice versa. The extent of variation among women in the heterogeneity terms is identified by multiple occurrences of each outcome for some women (births of different parity; different levels of education). Moreover, the observation of repeated events for a subset of women, with most women experiencing events belonging to both processes, means that identification is possible without covariate exclusions (Lillard, 1993; Upchurch et al., 2002).

## Results

In Table 3, we present the estimates of a standard event history model in which birth rates are modelled separately for each parity (Models 1 and 2) and the results when the equations for first, second and third births are estimated jointly (Models 3 and 4). The results when the fertility equations are modelled jointly with educational attainment are presented in Table 7 in annex (Models 5 and 6). As can be seen at the bottom of Table 7, the standard deviations of the heterogeneity terms for the fertility (0.52) and educational attainment (2.74) processes are statistically significant in both Models 5 and 6 (*p* < 0.01). This indicates that indeed there are selection effects influencing fertility. Yet, the correlation between the heterogeneity terms is not statistically significant, suggesting that there is no spurious relationship between education and fertility and that the fertility models capture the essential factors affecting fertility. Unsurprisingly, the correlation between the heterogeneity terms is highly sensitive to the variables included in the models. For instance, the inclusion of the fertility policy variable in the fertility equations led to a change in the correlation from significantly negative (− 0.13, *p* < 0.05) to a non-significant negative correlation, suggesting that this variable not only influences fertility, but also moderates the effect of educational attainment on fertility (which is the basis for our Hypothesis 5). Similarly, and consistently with our theoretical expectations, if family background factors are removed from the fertility equations in Models 5 and 6, we find a strong negative correlation between the processes (− 0.18, *p* < 0.01) and a larger effect of education on fertility, highlighting the importance of including these factors common to the educational and fertility processes to obtain unbiased estimates. It should be emphasized, however, that the effects of education for second and third births remain strongly negative and highly statistically significant irrespective of the specification. Given that the lack of statistical significance of the correlation between the residuals for fertility and education, we will no longer discuss the results in Table 7. The presentation that follows will mainly focus on Models 3 and 4, in which birth rates are modelled jointly. Note, however, that these results are similar to the ones obtained with the birth rates modelled separately for each parity.

**Fig. 2:**
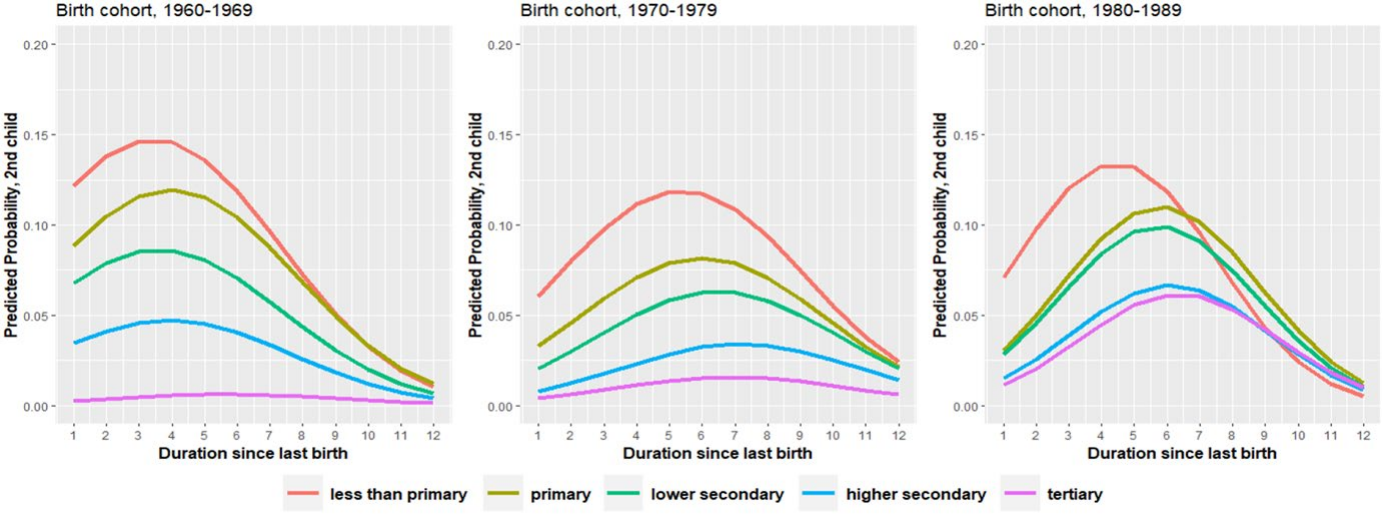
Predicted annual probability of a second birth by duration since first birth and women’s educational level, for each birth-cohort group. The model also includes controls for age at first birth, mother’s education, and fertility policy

About 55 percent of all women of the cohorts born in the 1960s and 1970s had a second child while, remarkably, this proportion increased to more than 70 percent in the 1980s birth-cohorts (Fig. 1). The survivor functions also show that second births’ progression ratios follow a strong educational gradient: while more than 76 percent of women with less than primary education bear a second child 15 years after bearing the first child, only about 26 percent of women with tertiary education bear a second child at the same duration. Multivariate results show that the relative risks are about 79 percent lower for the tertiary educated (Model 3, Table 3) with respect to the “less than primary” group and highly statistically significant (0.30/1.40 = 0.214; 1−0.214 = 0.79). The inclusion of an interaction between women’s educational level and the duration since first birth is statistically significant (Model 4), although the differentials in the timing are minor, affecting especially the “less than primary” group, which shows an earlier timing. Nevertheless, there is a clear and strong gradation in second-birth rates by level of education, irrespective of the duration since first birth (Fig. 2).

A similarly strong educational gradient is present for third births, although the proportion of women progressing to this parity is about 24 percent only, according to the survivor function’s results. This fact, together with the small educational differentials in the probability of first birth, highlights the key role of second births in explaining overall fertility levels for the studied birth-cohorts. Once again, some relatively small timing differentials by education are found for third births, which are statistically significant for the “less than primary” group. To our knowledge, no previous research has reported these timing differentials for second and third births for Chinese data. Highly educated individuals may decide to widen birth intervals to spread the costs of children over time. Additionally, they are likely to experience a steeper increase in their income by age, providing further incentives to delay subsequent births to a later period when they will earn higher incomes. Other possible explanations for the educational differentials in the timing of second and third births are a lower control of contraception by the least educated, and a possible insufficient control for timing polices in our models. Overall, these results give clear support to our first hypothesis: *Women’s educational attainment has a strong negative effect on the hazard of transition to a second or third child.*

Our second hypothesis states that the *male partner’s educational attainment has a negative effect on the hazard of a second or third birth.* Here the results are less extreme, albeit the educational differentials are still substantial (Table 4). Couples in which the man is tertiary educated display a risk of second birth 36 percent lower than the “less than primary” educated (0.70/1.09 = 0.64; 1−0.64 = 0.36). Given the small sample size for the highly educated at risk of a third child, we grouped the men with low secondary education and above in the models, which show a non-significant coefficient with respect to the primary educated. By contrast, the “less than primary educated” show a relative risk of about 20 percent higher than primary educated men.

In Table 5 we show interaction effects between the woman’s level of education and the birth cohort, for second and third births. The second birth relative risk differentials between educational levels sharply decline across birth cohorts. If the “less than primary” educated women of the 1960–69 birth cohorts are taken as the reference category, the relative risk for the tertiary educated is 0.03 (0.07/2.14 = 0.03), i.e. the risk is reduced by a factor 96 percent. But if the same calculation is made for the 1980–89 birth cohorts we find a relative risk for the tertiary educated of 0.25 (0.48/1.93 = 0.25), i.e. 75 percent lower. To further explore the changes over time of educational differentials we plotted the predicted probabilities of a second birth by duration since first birth separately for each birth cohort (Fig. 2).^7^ The results show that the convergence between educational levels was mainly achieved by a large increase in the second birth probabilities of the higher secondary and especially tertiary educated women. Second birth probabilities of lower educated women show a decline and some postponement in the cohorts born in the 1970s, relative to the ones born in the 1960s, while a substantial recovery is visible for women of all educational levels in the 1980s birth-cohort. These results for second births are clearly consistent with our third hypothesis, i.e*. the negative effect of education on second and third birth rates declines across birth cohorts*. Yet the evidence does not give support for such a decline in the case of third births, maybe because the risk of having the third birth is itself already very low (the likelihood ratio test comparing a model with and without the interaction was not statistically significant). The educational differentials actually increased across birth-cohorts for third births. For instance, the relative risk for women with higher secondary or tertiary education was 0.47 compared with the less than primary educated in the 1960–69 birth cohort, while the corresponding ratio was 0.29 for the youngest birth cohort.

Figure 1 shows that bearing a first birth is almost a universal behaviour for Chinese women, although a modest increase in childlessness is visible for the youngest cohort, i.e. those born in the 1980s. Women with higher secondary or tertiary education show slightly higher levels of childlessness (about 10 percent, with a confidence interval of 0.08–0.13) compared to women with the lowest level of education (about 5 percent, c.i.: 0.05–06) and a substantially delayed first birth timing: there is a 5-year differential between extreme educational groups in the median age at first birth. Such levels of childlessness and postponement are still limited in comparison to Japan, South Korea, or Taiwan (Jones, 2007; Raymo et al., 2015a, 2015b). The multivariate results presented in Table 3 specify these results. Being enrolled in education reduces the rate of first birth by more than 5 times (the relative risk of being enrolled in education versus not being enrolled in education is 0.20 at *p* < 0.01). The main effect of education is negative, since the relative risk of the tertiary educated is about half of the primary educated (0.46 at *p* < 0.01) (Model 3), but there is a significant interaction with age (Model 4). At ages below 26, there is a negative effect of education, which is largely compensated by the higher rates of women with higher secondary and tertiary education after that age. Overall, these results are consistent with Hypothesis 4 which stated that *irrespective of the educational level, most women bear at least one child* and that *the effect of education on first childbearing is limited to its postponement*.

As expected, the main effects of the fertility policy variable show a substantial negative effect for both second (relative risk: 0.67 at *p* < 0.01) and third births (r.r.: 0.83) (Model 4), albeit it is not statistically significant for third births (probably linked to the low number of observations for which third births were allowed). To investigate whether *the negative effect of fertility policies was stronger for the highly educated* (Hypothesis 5), we computed an interaction between the policy and education variables. This interaction yielded statistically significant results for second births, but not for third births. As shown in Table 6, the negative effect of the policy on the hazard of second births gradually becomes stronger with the level of education. There is a small differential between women who are not allowed to bear a second child compared to women who are allowed for women with “less than primary” education (1.37/1.57 = 0.87; 1−0.87 = 0.13; *p* < 0.01); but tertiary educated women subject to the policy show a relative risk 79 percent lower (0.12/0.58 = 0.21; 1−0.21 = 0.79; *p* < 0.01) than tertiary educated women who are allowed to bear a child.

**Table 3:** Estimates from separate and joint models for the processes of first, second and third birth (hazard models)

	Birth rates modeled separately for each parity		Birth rates modeled jointly, with a common unobserved factor	
	Model without interaction	Model with interaction	Model without interaction	Model with interaction
	Model 1	Model 2	Model 3	Model 4
**First birth**				
Age spline^a^				
15–20 years	0.72***	0.75***	0.74***	0.75***
20–23 years	0.28***	0.29***	0.34***	0.30***
23–26 years	0.05***	0.00	0.12***	0.02
26–30 years	− 0.13***	− 0.14***	− 0.08***	− 0.13***
30–45 years	− 0.21***	− 0.20***	− 0.20***	− 0.19***
Woman’s education^b^				
Less than primary	0.99	0.85***	1.02	0.85***
Primary (ref.)	1	1	1	1
Low secondary	0.85***	0.96	0.81***	0.95
High secondary	0.64***	0.84***	0.56***	0.81***
Tertiary	0.59***	0.47***	0.46***	0.44***
Woman’s education additional effect at age < 23^b^				
Less than primary		1.33***		1.33***
Low secondary		0.83***		0.84***
High secondary		0.50***		0.50***
Tertiary		0.71***		0.73***
Woman’s education additional effect at age > 26^b^				
Less than primary		0.88*		0.87*
Low secondary		0.88**		0.88**
High secondary		1.11		1.10
Tertiary		2.25***		2.22***
Not enrolled in education	1	1	1	
Enrolled in education^b^	0.18***	0.27***	0.20***	0.27***
Mothers’ education^b^				
Less than primary	0.98	0.98	0.98	0.98
Primary (ref.)	1	1	1	1
Low secondary	0.94*	0.94**	0.93*	0.93**
High secondary or tertiary	0.86***	0.84***	0.83***	0.84***
Parental occupation^b^				
Agriculture	1.07*	1.06	1.07	1.06
Unskilled (ref.)	1	1	1	1
Skilled	1.00	0.99	1.00	0.99
Services	0.99	0.98	0.97	0.98
Professional	1.02	1.01	1.01	1.01
Birth cohort^b^				
1960–69	1.07***	1.07***	1.05**	1.07***
1970–79 (ref.)	1	1	1	1
1980–89	0.88***	0.88***	0.84***	0.87***
No siblings^b^	0.88***	0.87***	0.85***	0.86***
1 sibling (ref.)	1	1	1	1
2 + siblings	1.02	1.00	0.98	1.00
Household registration				
Rural hukou (ref.)	1	1	1	1
Urban hukou	0.81***	0.81***	0.82***	0.80***
Constant term	− 5.61***	− 5.67***	− 5.71***	− 5.69***
**Second birth**				
Duration spline^a^				
0–1 years	4.46***	4.37***	4.45***	4.37***
1–3 years	0.12***	0.18***	0.17***	0.19***
3–6 years	− 0.13***	− 0.11***	− 0.11***	− 0.10***
6 + years	− 0.17***	− 0.17***	− 0.16***	− 0.17***
Woman’s education^b^				
Less than primary	1.35***	1.68***	1.40***	1.69***
Primary (ref.)	1	1	1	1
Low secondary	0.76***	0.83***	0.71***	0.82***
High secondary	0.54***	0.52***	0.46***	0.50***
Tertiary	0.39***	0.29***	0.30***	0.28***
Woman’s education additional effect at duration > 3^b^				
Less than primary		0.68***		0.69***
Low secondary		0.88***		0.87**
High secondary		1.06		1.04
Tertiary		1.49***		1.46**
Age at first birth^b^				
< 20	1.34***	1.33***	1.25***	1.32***
20–22	0.78***	0.78***	0.91**	0.81***
23–25(ref.)	1	1	1	1
26–28	0.71***	0.72***	1.01	0.77***
> 28	0.40***	0.40***	0.67***	0.45***
Mothers’ education^b^				
Less than primary	1.30***	1.29***	1.32***	1.30***
Primary (ref.)	1	1	1	1
Lower secondary	0.88**	0.88***	0.85***	0.87***
Higher secondary or tertiary	0.92	0.93	0.89	0.92
Policy allows second child (ref.)	1	1	1	1
Policy does not allow^b^ second child	0.67***	0.67***	0.66***	0.67***
Constant term	− 6.46***	− 6.50***	− 6.68***	− 6.55***
**Third birth**				
Duration spline^a^				
0–1 years	3.26***	3.20***	3.27***	3.20***
1–3 years	0.04	0.09	0.06	0.10
3–5 years	− 0.64***	− 0.58***	− 0.63***	− 0.57***
5 + years	− 0.27***	− 0.28***	− 0.27***	− 0.28***
Woman’s education^b^				
Less than primary	1.34***	1.53***	1.40***	1.55***
Primary (ref.)	1	1	1	1
Low secondary	0.84**	0.90	0.79***	0.90
High secondary or tertiary	0.64***	0.61***	0.54***	0.59***
Woman’s education additional effect at duration > 3 ^b^				
Less than primary		0.73***		0.73***
Low secondary		0.85		0.84
High secondary or tertiary		1.13		1.13
Age at second birth^b^				
< 23	1.49***	1.49***	1.23***	1.44***
23–28 (ref.)	1	1	1	1
> 28	0.39***	0.39***	0.48***	0.41***
Mothers’ education^b^				
Less than primary	1.32***	1.33***	1.38***	1.34***
Primary (ref.)	1	1	1	1
Secondary or tertiary	1.13	1.13	1.08	1.12
Policy allows third child (ref.)	1	1	1	1
Policy does not allow third child^b^	0.84	0.84	0.81	0.83
Constant term	− 6.03***	− 6.07***	− 6.32***	− 6.14***
Heterogeneity components				
Standard deviation (fertility) ɛ			0.52***	0.23***
Log-likelihood	− 108,172	− 107,878	− 108,132	− 107,879

Based on data from the China Family Panel Studies (2010–18) for women born between 1960 and 1989

Significance: ‘*’ = 10%; ‘**’ = 5%; ‘***’ = 1%. ^*a*^Slope parameters. ^*b*^Relative risks (exponentiated coefficients)

**Table 4:** Relative risks of a second birth by spouse’s education

	Second births		Third birth	
	Risk ratios	Sig.	Risk ratios	Sig.
Spouse’s education				
Less than primary	1.09	**	1.20	**
Primary (ref.)	1		1	
Low secondary	0.95		0.93	
High secondary	0.86	***	1.06	
Tertiary	0.70	***		
Births	6,189		1,375	
Women	11,354		6,258	

Estimates based on data from the China Family Panel Studies 2010–18

Specification as in Model 4. For third births the “low secondary” category includes higher educational levels. Significance: ‘*’ = 10%; ‘**’ = 5%; ‘***’ = 1%

**Table 5:** Relative risks of a second or third birth by birth cohort

Woman’s education	Birth-cohort		
	1960–69	1970–79	1980–89
**Second birth**			
Less than primary	2.14***	1.83***	1.93***
Primary	1.38***	1 (ref.)	1.15*
Low secondary	0.96	0.80***	1.15*
High secondary	0.49***	0.44***	0.81*
Tertiary	0.07***	0.16***	0.48***
**Third birth**			
Less than primary	1.86***	1.44***	1.71***
Primary	1.19	1 (ref.)	0.95
Low secondary	1.14	0.70**	0.96
High secondary or tertiary	0.87	0.47**	0.49**

Estimates based on data from the China Family Panel Studies 2010–18

Specification as in Model 4. Significance: ‘*’ = 10%; ‘**’ = 5%; ‘***’ = 1%

**Table 6:** Relative risks of a second birth: interaction between education and fertility policy

	Fertility policy	
	2nd child allowed	2nd child not allowed
Woman’s educational level		
Less than primary	1.57***	1.37**
Primary	1 (ref.)	0.80***
Low secondary	1.00	0.59***
High secondary	0.86	0.31***
Tertiary	0.58***	0.12***

Estimates based on data from the China Family Panel Studies 2010–18

Specification as in Model 4. Significance: ‘*’ = 10%; ‘**’ = 5%; ‘***’ = 1%

## Discussion and conclusions

China’s fertility levels during the Reform Era (1980–2018) have been surprisingly low, considering its levels of income, urbanisation, or agricultural labour force. Even compared to other East Asian societies, China reached very low fertility levels at an earlier stage in economic development (Raymo et al., 2015a, 2015b). The role of the massive economic, institutional and cultural changes in bringing about very low levels of fertility has been intensely debated, especially with respect to the relative importance of fertility policies (Cai, 2010; Feng, 2015; Goodkind, 2017; Zhao & Zhang, 2018). While several previous contributions have focused on the effect of macro-level indicators, here we have examined the individual-level impact of education on fertility during the Reform Era. Assessing this relationship provides some crucial micro-level foundations for understanding recent very low fertility levels. Our analyses contribute to the existing evidence showing that the size and sign of the association between education and fertility are highly context-specific (Bongaarts, 2003; Klesment et al., 2014; Lutz & Skirbekk, 2014; Yoo, 2014). Indeed, the results found bear some similarities (as well as some differences) with other societies in advanced stages of the fertility transition. Yet the specificities of the Chinese transition from a state-planned to a market economy sets China apart from the experience of other societies that made that transition.

One of the main contributions of this paper has been to develop a set of hypotheses that specify how the economic and institutional changes that took place during the Reform Era influenced the relationship between education and fertility. Drawing on institutional and gender theoretical perspectives, we have pointed to some key processes, such as the expansion of the market economy, the retreat of social policies providing economic security and support with the cost of children, and changes in fertility policies. The institutional setting provided incentives for a rapid increase in the levels of education for both genders and the labour force participation of most women of childbearing age. These conditions were conducive to a modicum of women’s autonomy, while substantial gender inequalities remain in the labour market and care obligations. Family economies still heavily rely on intense intergenerational exchanges. The strong educational layering in fertility behaviour that we found can be situated in this economic and institutional context.

A further contribution of this paper has been to provide detailed empirical analyses assessing the hypotheses proposed, leading to new insights into the relationship education-fertility. Overall, the results are highly consistent with the hypotheses proposed, providing support to institutional perspectives on fertility (McDonald, 1997; McNicoll, 2001; Rindfuss and Choe 2016). It should be emphasised that our estimates account for the influence of policies measured at the individual-level and control for an array of individual and family background variables that act as confounders in the relationship between education and fertility. Indeed, our results show that such controls are crucial to obtain unbiased estimates. Of course, future research may further investigate the role of variables that could not be included here due to the lack of data, such as the women’s family values and her parent’s resources, as well as an explicit inclusion of macro-level factors.^8^ A further limitation of the study concerns the measurement of fertility policies, that had to rely on several observed characteristics of the women and her partner, due to the lack of a direct measurement of the policies. The robustness of our results is reinforced by the use of event history models with simultaneous equations (to account for self-selection) and the test of a possible correlation between unmeasured attributes affecting both educational attainment and fertility (to account for reverse causality). Yet these models impose several assumptions, including that unobservables that affect both fertility and education are woman-specific and time invariant, and that they are jointly normally distributed. It should be noted, however, that the main results presented in the paper hold irrespective of the particular event-history model used to estimate the effects of education on fertility. Overall, our empirical strategy intended to disentangle causal effects from selection effects. One way of assessing the existence of a causal relationship between education and fertility is by using the three criteria proposed by Lutz and Skirbekk (2014; see also Ní Bhrolcháin & Dyson, 2007): First, we found *a strong association* between education and fertility at the individual level, using life course data. Second, existing theories offered a *plausible narrative about the mechanisms* through which education influences fertility. And third, *other competing explanations of the observed association could be ruled out as playing a dominant role*, particularly the influence of self-selection and reverse causality.

The results point to a substantial contribution of the increase in the educational attainment of the population in the fertility decline and current very low fertility levels. In particular, bearing a second, and to a lesser extent a third birth, shows a neat negative association with education. These results are consistent with educational differentials in social mobility opportunities for both, parents and their child(ren) and the related differentials in the costs of rearing children. This is especially so in a context with high returns to education, weak social-support policies, and increasing socio-economic inequalities. Economic security considerations together with low investments in children are likely to dominate fertility decisions among low educated parents. By contrast, increased parental education should involve heightened economic and social costs of children, especially for women. These sets of factors bear clear similarities with those prevalent in other East Asian societies with very low fertility, that share with China several institutional and cultural characteristics (Gietel-Basten, 2019; Jones et al., 2009; Zhao & Zhang, 2018). Remarkably, not only women’s education but also men’s education leads to lower fertility, consistent with the above interpretation. While theoretical expectations on the effect of men’s education are ambiguous, previous empirical evidence (mostly focusing on highly developed countries) shows a positive effect (Klesment et al., 2014). Our analyses suggest that the higher purchasing power of better educated men is outbalanced by higher child-quality requirements and material expectations. This result is likely to occur in countries in which most of the costs of children fall on parents, in which a high educational competition is prevalent, and with a familistic welfare regime.

We also hypothesised that the vast majority of women, irrespective of their educational level, bear at least one child, and this was corroborated by our results. In a context with a strong economic and social interdependence between generations, there are compelling incentives for marriage and bearing at least one child. As a result, child(ren)’s future socio-economic position matters for the parents, reinforcing the need for child investments. Our results confirmed the hypothesis of a decline in the negative effect of education for second births across the cohorts born in the 1970s and especially 1980s, compared with the 1960s birth-cohorts. Yet the results for third birth did not support our hypothesis, highlighting that third births continue to be confined to the (decreasing proportion of the) very low educated. These results extend to more recent birth cohorts the previous finding of a widening of educational differentials between the cohorts born in the 1940s to the mid1960s, i.e. during the central stages of the demographic transition (Niu & Qi, 2020; Piotrowski & Tong, 2016). A gradual convergence between educational groups is likely to be the result of the changes that took place during the Reform Era, including a weakening in the role of children as labour and economic security providers among the low educated, the generalised increase in educational attainment, and the spread of family norms favouring the one-child family from highly educated parents to lower educational groups. It is remarkable that the highly educated members of the 1980s birth cohort sharply increased their second birth rates, while lower educated women showed weaker increases. This birth cohort experienced major contextual transformations during their central reproductive years, resulting from the acceleration of social welfare and market reforms. Since the late 1990s, it took place a rapid expansion of tertiary education and substantial income increases, together with a rise in the returns to education, and a sharp increase in labour market informality and job insecurity (which especially hit the low educated) (Jansen & Wu, 2012; Li, 2013). An additional change that may help explain the increase in second birth rates by the better educated is the gradual relaxation of the one child policy, leading to its abolition in 2016. The weakening of norms prescribing one child, beyond actual policy rules, is likely to have affected disproportionately the highly educated.

A crucial component of the institutional setting is the existence of a stringent fertility policy based on a strong political and administrative structure. This policy stipulated different family sizes according to the specific socio-economic situation of individuals, thus already implying some degree of educational stratification in fertility. Not surprisingly, our results show a substantial effect of policies on both second and third births, implying a reduction of about one third in the hazard of second births and of about 17 percent in third births. Moreover, we hypothesise that compliance with the policy involved economic and social benefits that were positively stratified by level of education, while the low educated had higher incentives for bearing second and third births and lower penalties associated with contravening the policy. Indeed, our results show that the effect of fertility policies on second births was substantially more negative for the highly educated, while they did not show significant results for third births. To our knowledge, the results reported here are the first ones specifying the effect of fertility policies at the individual level during the whole one-child policy period, using life course data. Even if data availability constraints may lead to some underestimation of the policy effects, it seems clear that they did not preclude the (much stronger) effects of education and other individual-level socio-economic variables. Beyond direct policy effects, it is likely that the existence of powerful policies regulating marriage and fertility for more than four decades shaped norms about family life, including child investments and women’s labour force participation. This can result from a reciprocal interaction between ideal and actual family size. Moreover, the institutional context was increasingly geared toward the one-child family, particularly concerning the educational system and labour market organisation, which also helps to explain why the policy was widely accepted. This suggests that the institutional configuration that was created during the one-child policy period continues to influence current parents’ fertility choices.

## Data Availability

The data that support the findings of this study are openly available at “China Family Panel Studies (CFPS)” at https://doi.org/10.18170/DVN/45LCSO by the Institute of Social Science Survey, Peking University, 2015.

## Appendix

See Table

**Table 7:** Estimates from joint models for the processes of first, second and third birth (hazard models), and for educational attainment (probit model)

	Model 5			Model 6		
	Model without interaction			Model with interaction		
	Coef.	Risk ratio	Sign.	Coef.	Risk ratio	Sign.
**First birth**						
Age spline (slope parameters)						
15–20 years	0.74		***	0.75		***
20–23 years	0.34		***	0.30		***
23–26 years	0.12		***	0.02		
26–30 years	− 0.08		***	− 0.13		***
30–45 years	− 0.20		***	− 0.19		***
Woman’s education						
Less than primary	0.01	**1.01**		− 0.20	**0.82**	***
Primary (ref.)	0	**1**		0	**1**	
Low secondary	− 0.21	**0.81**	***	− 0.02	**0.98**	
High secondary	− 0.57	**0.57**	***	− 0.14	**0.87**	**
Tertiary	− 0.76	**0.47**	***	− 0.71	**0.49**	***
Woman’s education additional effect at age < 23						
Less than primary				0.29	**1.33**	***
Low secondary				− 0.18	**0.84**	***
High secondary				− 0.69	**0.50**	***
Tertiary				− 0.34	**0.71**	***
Woman’s education additional effect at age > 26						
Less than primary				− 0.14	**0.87**	*
Low secondary				− 0.13	**0.88**	**
High secondary				0.10	**1.10**	
Tertiary				0.80	**2.22**	***
Not enrolled in education (ref.)	0	**1**		0	**1**	
Enrolled in education	− 1.61	**0.20**	***	− 1.27	**0.28**	***
Mothers’ education						
Less than primary	− 0.02	**0.98**		0.00	**1.00**	
Primary (ref.)	0	**1**		1	**1**	
Low Secondary	− 0.08	**0.92**	*	− 0.08	**0.92**	**
High Secondary or tertiary	− 0.19	**0.83**	***	− 0.20	**0.82**	***
Parental occupation						
Agriculture	0.07	**1.07**		0.07	**1.07**	*
Unskilled (ref.)	0	**1**		1	**1**	
Skilled	0.00	**1.00**		− 0.01	**0.99**	
Services	− 0.03	**0.97**		− 0.03	**0.97**	
Professional	0.01	**1.01**		0.01	**1.01**	
Birth cohort						
1960–69	0.05	**1.05**	**	0.08	**1.08**	***
1970–79 (ref.)	0	**1**		0	**1**	
1980–89	− 0.17	**0.84**	***	− 0.15	**0.86**	***
No siblings	− 0.16	**0.85**	***	− 0.15	**0.86**	***
1 sibling (ref.)	0	**1**		0	**1**	
2 + siblings	− 0.02	**0.98**		0.00	**1.00**	
Household registration						
Rural hukou (ref.)	0	**1**		0		
Urban hukou	− 0.20	**0.82**	***	− 0.22	**0.80**	***
Constant term	− 5.72		***	− 5.72		***
**Second birth**						
Duration spline (slope parameters)						
0–1 years	4.45		***	4.37		***
1–3 years	0.17		***	0.19		***
3–6 years	− 0.11		***	− 0.10		***
6 + years	− 0.16		***	− 0.17		***
Woman’s education						
Less than primary	0.34	**1.40**	***	0.49	**1.63**	***
Primary (ref.)	0	**1**		0	**1**	
Low secondary	− 0.33	**0.72**	***	− 0.17	**0.84**	***
High secondary	− 0.78	**0.46**	***	− 0.63	**0.53**	***
Tertiary	− 1.20	**0.30**	***	− 1.19	**0.31**	***
Woman’s education additional effect at duration > 3						
Less than primary				− 0.38	**0.68**	***
Low secondary				− 0.14	**0.87**	**
High secondary				0.04	**1.04**	
Tertiary				0.39	**1.47**	**
Age at first birth						
< 20	0.22	**1.25**	***	0.28	**1.32**	***
20–22	− 0.09	**0.91**	*	− 0.22	**0.81**	***
23–25 (ref.)	0	**1**		0	**1**	
26–28	0.01	**1.01**		− 0.26	**0.77**	***
> 28	− 0.39	**0.68**	***	− 0.80	**0.45**	***
Mothers’ education						
Less than primary	0.29	**1.34**	***	0.28	**1.33**	***
Primary (ref.)	0	**1**		0	**1**	
Lower secondary	− 0.16	**0.85**	***	− 0.15	**0.86**	***
Higher secondary or tertiary	− 0.12	**0.89**		− 0.11	**0.89**	
Policy allows second child (ref.)	0	**1**		0	**1**	
Policy does not allow second child	− 0.41	**0.66**	***	− 0.40	**0.67**	***
Constant term		**1.00**	***	− 6.58		***
**Third birth**						
Duration spline (slope parameters)						
0–1 years	3.26		***	3.20		***
1–3 years	0.06			0.10		*
3–5 years	− 0.63		***	− 0.57		***
5 + years	− 0.27		***	− 0.28		***
Woman’s education						
Less than primary	0.34	**1.40**	***	0.40	**1.49**	***
Primary (ref.)	0	**1**		0	**1**	
Low secondary	− 0.23	**0.79**	***	− 0.08	**0.92**	
High secondary or tertiary	− 0.61	**0.54**	***	− 0.46	**0.63**	**
Woman’s education additional effect at duration > 3						
Less than primary				− 0.31	**0.73**	***
Low secondary				− 0.17	**0.84**	
High secondary or tertiary				0.12	**1.13**	
Age at second birth						
< 23	0.21	**1.23**	***	0.37	**1.45**	***
23–28 (ref.)	0	**1**		0	**1**	
> 28	− 0.73	**0.48**	***	− 0.90	**0.41**	***
Mothers’ education						
Less than primary	0.32	**1.38**	***	0.31	**1.37**	***
Primary (ref.)	0	**1**		0	**1**	
Secondary or tertiary	0.08	**1.08**		0.09	**1.10**	
Policy allows third child (ref.)	0	**1**		0	**1**	
Policy does not allow third child	− 0.21	**0.81**		− 0.18	**0.83**	
Constant term	− 6.32		***	− 6.16		***
**Progression to the next educational level**						
Mothers’ education						
Less than primary	− 1.38		***	− 1.15		***
Primary (ref.)	0			0		
Lower secondary	0.83		***	0.69		***
Higher secondary or tertiary	1.90		***	1.57		***
Previous educational level						
Primary	1.95		***	1.57		**
Low secondary (ref.)	0			0		
High secondary	− 2.80		***	− 2.35		***
College	− 4.56		***	− 3.74		***
University	− 6.03		***	− 4.94		***
Birth cohort						
1960–64	0.50		***	0.43		**
1965–69 (ref.)	0			0		
1970–74	0.33		**	0.27		**
1975–79	1.32		***	1.08		***
1980–84	2.07		***	1.72		***
1985–89	2.58		***	2.14		***
Parental occupation						
Agriculture	− 0.63		***	− 0.51		**
Unskilled (ref.)	0			0		
Skilled	0.30		*	0.25		*
Services	0.36		**	0.29		*
Professional	0.29		*	0.25		*
No siblings	0.18			0.14		
1 sibling (ref.)	0					
2 siblings	− 0.22		**	− 0.18		*
3 siblings	− 0.27		**	− 0.22		**
4 + siblings	− 0.70		***	− 0.56		***
Parent communist party member	0.63		***	0.52		***
Ethnic minority	− 1.30		***	− 1.07		***
Constant term	0.78		***	0.68		***
Heterogeneity components						
Standard deviation (fertility) ɛ	0.52		***	0.23		***
Standard deviation (education) λ	2.74		***	2.20		**
Correlation ɛ λ	− 0.01			− 0.20		
Log-likelihood	− 127,224			− 126,968		

Significance: ‘*’ = 10%; ‘**’ = 5%; ‘***’ = 1%. Risk ratios are in bold.

Based on data from the China Family Panel Studies (2010–18) for women born between 1960 and 1989

^7^.

## Footnotes

1 Different methods have been used in the literature to account for unobserved factors, including quasi-experimental methods exploiting educational reforms (Sohn and Lee 2019) or the use of twin models (Kohler et al., 1999).

2 Female labour force participation rate was 73% in 1990 and declined to 61% in 2018 (International Labour Organization 2021).

3 Currently paternity leave has a duration of 14–30 days. Leave regulations depend on provincial authorities and are mainly relevant for formal sector employees, thus marginalising the large informal sector and the self-employed in agriculture.

4 The data are openly available at https://doi.org/10.18170/DVN/45LCSO.

5 Impicciatore and Dalla Zuanna (2017) adopted a similar approach to study the impact of educational attainment on fertility in Italy; yet they used an ordered probit to model educational attainment, which, contrary to our specification, did not allow to estimate the standard deviation of the heterogeneity component of education. Different educational decisions belonging to the same women are unlikely to be independent, and our specification accounts for that correlation. Moreover, using a simple probit model facilitates the interpretation of the results**.** Our approach is also similar to the one followed by Billari and Philipov (2004) and Martin-Garcia and Baizan (2006) to study the interrelationship between first births and educational attainment.

6 The variables included in the model are: mother’s education, birth cohort, parental occupation, number of siblings, whether one of the parents is member of the communist party; ethnic minority membership, and level of education attained by the woman. This last variable controls for the very different probability of attaining an additional level of education for women in each level of education.

7 In Fig. 2 we use a discrete-time model that allows the inclusion of the time since first birth as a continuous variable (years since first birth and years since first birth squared, and their interaction with education). The model also includes controls for age at first birth, mother’s education, and whether the policy allows a second birth.

8 The empirical analysis of the link between contextual variables and fertility was outside the scope of this paper.
